# Removal of Microcystin-LR by a Novel Native Effective Bacterial Community Designated as YFMCD4 Isolated from Lake Taihu

**DOI:** 10.3390/toxins10090363

**Published:** 2018-09-08

**Authors:** Fei Yang, Jian Guo, Feiyu Huang, Isaac Yaw Massey, Ruixue Huang, Yunhui Li, Cong Wen, Ping Ding, Weiming Zeng, Geyu Liang

**Affiliations:** 1 Department of Occupational and Environmental Health, Xiangya School of Public Health, Central South University, 110 Xiangya Road, Changsha 410078, China; guojianph@csu.edu.cn (J.G.); huangfeiyu@csu.edu.cn (F.H.); robert@csu.edu.cn (I.Y.M.); wencong941017@csu.edu.cn (C.W.); yixp176911007@csu.edu.cn (P.D.); 2 Key Laboratory of Environmental Medicine Engineering, Ministry of Education, School of Public Health Southeast University, Nanjing 210009, China; yhli@seu.edu.cn (Y.L.); gyliang@seu.edu.cn (G.L.); 3 Key laboratory of Hunan Province for Water Environment and Agriculture Product Safety, Central South University, Changsha 410083, China; 4 Key Laboratory of Biometallurgy, Ministry of Education, School of Minerals Processing and Bioengineering, Central South University, Changsha 410083, China; hyacinth_hai@csu.edu.cn

**Keywords:** microcystin-LR (MC-LR), remove, Adda, bacterial community

## Abstract

Microcystin-LR (MC-LR) is the most toxic and frequently detected monocyclic heptapeptide hepatotoxin produced by cyanobacteria, which poses a great threat to the natural ecosystem and public health. It is very important to seek environment-friendly and cost-efficient methods to remove MC-LR in water. In this study, the MC-degrading capacities of a novel indigenous bacterial community designated as YFMCD4 and the influence of environmental factors including various temperatures, MC concentrations and pH on the MC-degrading activities were investigated utilizing high-performance liquid chromatography (HPLC). In addition, the MC-degrading mechanism of YFMCD4 was also studied using HPLC coupled with a mass spectrometry equipped with electrospray ionization interface (HPLC-ESI-MS). The data showed MC-LR was completely removed at the maximum rate of 0.5 µg/(mL·h) under the optimal condition by YFMCD4. Two pure bacterial strains *Alcaligenes faecalis* and *Stenotrophomonas acidaminiohila* were isolated from YFMCD4 degraded MC-LR at a slower rate. The MC-degrading rates of YFMCD4 were significantly affected by different temperatures, pH and MC-LR concentrations. Two intermediates of a tetrapeptide and Adda appeared in the degradation process. These results illustrate that the novel YFMCD4 is one of the highest effective MC-degrading bacterial community, which can completely remove MC-LR and possesses a significant potential to treat water bodies contaminated by MC-LR.

## 1. Introduction

Cyanobacterial harmful algal blooms (CyanoHABs) have proliferated worldwide because of eutrophication and climate change [1,2,3,4]. Microcystins (MCs) produced by *Microcystis*, *Anabaena*, *Oscillatoria*, and *Nostoc* during CyanoHABs threaten public health and have become a serious global problem due to their extreme toxicities, which have attracted global attention [3,5]. MCs are a group of monocyclic heptapeptide hepatotoxins with a common genetic structure cyclo-(d-Ala-X-d-MeAsp-Z-Adda-d-Glu-Mdha-), where X and Z represent variable l-amino acids, and Adda is the b-amino acid residue of 3-amino-9-methoxy-2,6,8-trimethyl-10-phenyldeca-4,6-dienoic acid. Until now, over 100 analogs of MCs have been identified and MC-LR is the most toxic and abundant MC variant [6,7,8]. MC-LR is harmful to different organs including liver, intestine, colon, brain, kidney, lung, heart and reproductive system because it can inhibit the activities of protein phosphatases and affect the regulation of miRNA expression in these systems [9,10]. Even the chronic exposure to low concentrations of MCs can promote tumor growth [11]. The International Agency for Research on Cancer (IARC) has classified MC-LR as a possible carcinogen because of its potential carcinogenic activity [12]. To reduce MC-LR risks, the World Health Organization (WHO) has proposed a provisional guideline of 1 µg/L MCs in drinking water and this guideline level has been adopted in many countries such as USA, Australia, and China [13].

MC-LR is very stable and resistant to many natural factors including extreme pH, high temperature, and sunlight in the environment owing to its cyclic structure [3,6,14]. Moreover, MC-LR can be accumulated in aquatic organisms and food crops representing a health hazard to humans and animals through food chains [10,15,16]. It is very important to reduce MC-LR concentrations in freshwater ecosystem. However, conventional drinking water treatments have limited efficacy in removing MC-LR. Some physical and chemical methods containing ozonation, chlorination, photocatalysis, and electrolysis have been proposed for MC-LR elimination from drinking water. However, all these methods have certain limitations in terms of high operational costs, low efficacy and harmful by-products [3,6,17,18]. It is desirable that investigators seek other environmentally-benign and cost-efficient methods and technologies to remove MC-LR found in water bodies [3,6,17,18,19,20,21].

A few investigations demonstrated that microbial biodegradation may be one of the most environment-friendly, effective and promising treatment methods for removing MC-LR in natural waters, since it can detoxify MC-LR without generating any apparent potential harmful by-products [3,6,17,18,19,20]. Some MC-degrading pure bacterial strains have been isolated, identified, and had their mechanisms reported, where most of the isolated MC-degrading bacteria were limited to the family *Sphingomonadaceae* [19,20,22]. In practice, native bacterial communities (indigenous bacterial mixed culture) may be more suitable for degrading MC-LR in the environment compared to the single pure bacterial strains [6,23]. Therefore, it is important to obtain some native mixed bacterial communities for MC-LR removal.

Lake Taihu is the third largest lake in China with a total water surface area of about 2338 km^2^. Lake Taihu is essential to millions of people for drinking water, aquaculture, industrial activities, and recreation, but it has experienced CyanoHABs every year during the last three decades [3,6,18,22]. The MCs and odorous produced during CyanoHABs resulted in more than 2 million residents in Wuxi City being without drinking water for a week. Thus, it is desirable to obtain bacterial strains and remove MC-LR in water. In this study, the MC-LR removal capacities of a novel native bacterial community designated as YFMCD4 from Lake Taihu were determined under various environmental factors containing different temperatures and pH, as well as MC-LR concentrations. Moreover, the MC-removal mechanism, including the degradation pathway and products of YFMCD4, was also investigated.

## 2. Results 

### 2.1. Acquisition of Bacterial Community and Pure Bacterial Strains

**Figure 1:**
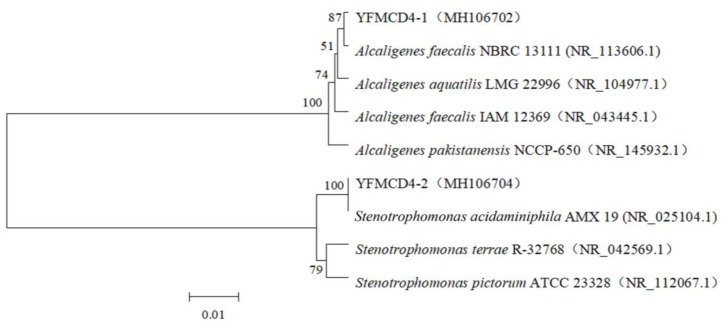
Construction of the phylogenetic tree based on the bacterial 16S rRNA gene sequences of the YFMCD4-1 and YFMCD4-2 using the neighbor-joining method.

A novel MC-degrading bacterial community, named YFMCD4, was obtained. Two pure bacterial strains designated YFMCD4-1 and YFMCD4-2 were isolated from the bacterial community YFMCD4 and identified according to 16S rRNA gene sequences. YFMCD4-1 and YFMCD4-2 were classified as *Alcaligenes faecalis* and *Stenotrophomonas acidaminiohila*, respectively (Figure 1). The nucleotide sequences of 16S rRNA genes from YFMCD4-1and YFMCD4-2 were deposited in the NCBI database with accession number MH106702 and MH106704, respectively. 

### 2.2. MC-Degrading Activities under Different Conditions

**Figure 2:**
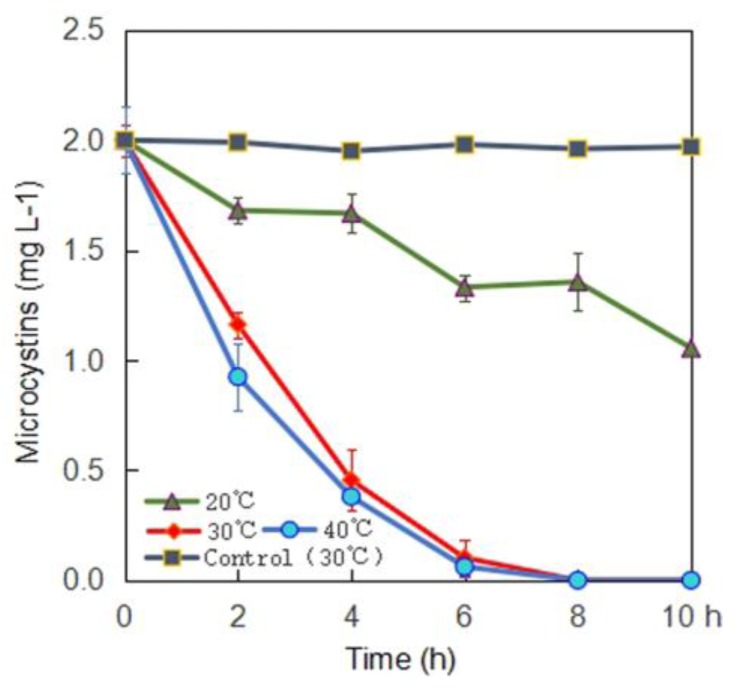
Effect of incubation temperature on the degradation rate of MC-LR by YFMCD4.

**Figure 3:**
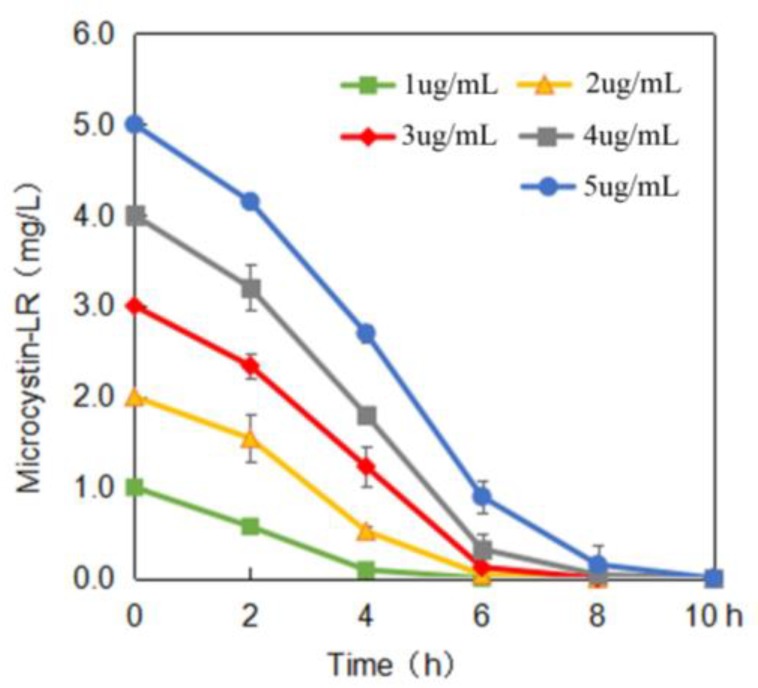
Effect of MC-LR concentration on the degradation of MC-LR by YFMCD4.

**Figure 4:**
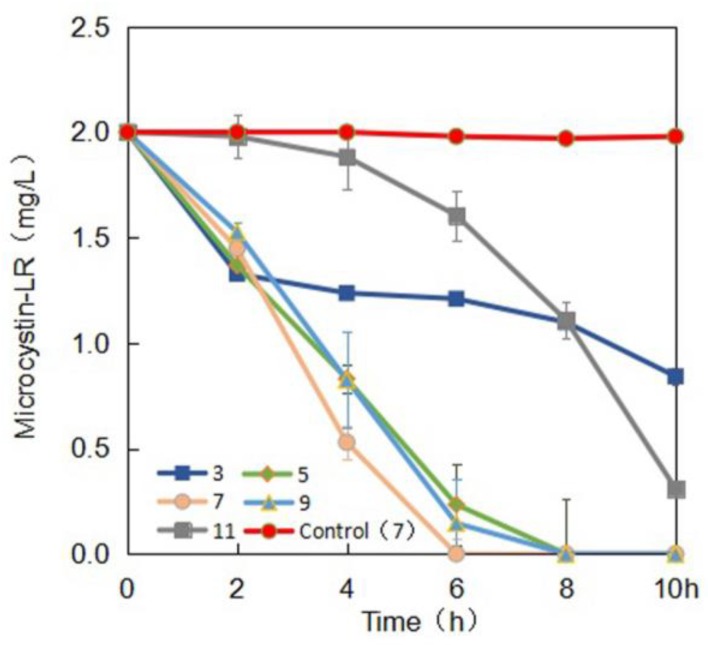
Effect of pH on the degradation of MC-LR by bacterial community YFMCD4.

Single-factor experiments were performed and the results are shown in Figure 2, Figure 3 and Figure 4. The MC-LR degrading rates of the bacterial community YFMCD4 were influenced by different incubation temperatures (Figure 2), MC-LR concentrations (Figure 3) and pH (Figure 4). Figure 2 showed that YFMCD4 degraded MC-LR at the average rate of 0.09, 0.33, 0.25 µg/(mL·h) at 20 °C, 30 °C, and 40 °C, respectively in 10 h.

Figure 3 illustrated that pH 7 and 30 °C MC-LR at concentrations of 1, 2, 3, 4, or 5 µg/mL were degraded at the average rate of 0.25, 0.33, 0.375, 0.5 and 0.5 µg/(mL·h) in 10 h, respectively. Figure 4 demonstrated that at 30 °C 2 µg/mL MC-LR was degraded by YFMCD4 at the average rate of 0.12, 0.25, 0.33, 0.25, and 0.17 µg/(mL·h) at pH 3, 5, 7, 9, and 11 in 10 h, respectively. The results indicated that the highest MC-degrading rate for YFMCD4 was 0.5 µg/(mL·h) at 30 °C and pH 7 with MC-LR concentrations of 4 or 5 µg/mL. It should be noted that there was no MC-LR degradation in the control media without bacterial community YFMCD4.

The two isolated pure bacteria strains YFMCD4-1 and YFMCD4-2 from YFMCD4 were used independently to degrade MC-LR. The results showed that within 10 h, YFMCD4-1 and YFMCD4-2 degraded 2 mg/L MC-LR up to 4.8% and 3.4% at a slower average rate of 0.96 × 10^−2^ µg/(mL·h) and 0.68 × 10^−2^ µg/(mL·h), respectively. Within five days, YFMCD4-1 and YFMCD4-2 further degraded MC-LR up to 17.0% and 18.0% also at a slower average rate of 0.28 × 10^−2^ µg/(mL·h) and 0.3 × 10^−2^ µg/(mL·h), respectively (Figure S1).

### 2.3. MC-LR Analysis and Degradation Products

**Figure 5:**
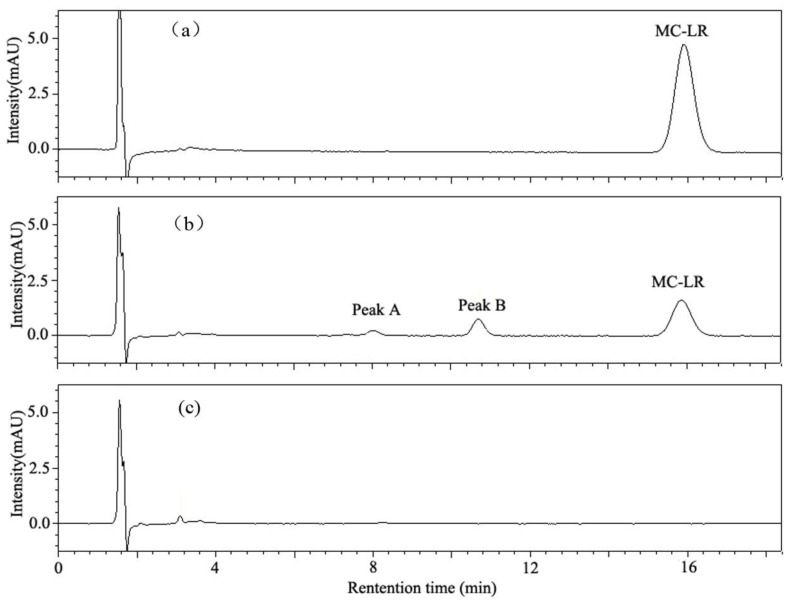
High-performance liquid chromatography (HPLC) chromatograms obtained during MC-LR degradation incubated with bacterial community YFMCD4 at time 0 h (**a**), 4 h (**b**), and 10 h (**c**).

**Figure 6:**
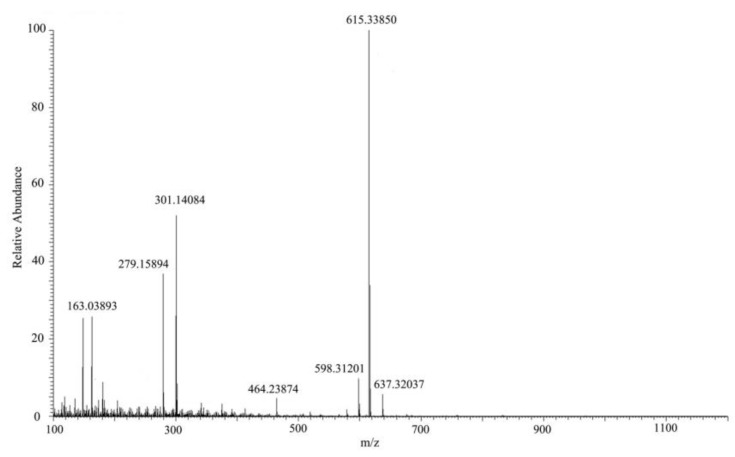
HPLC-ESI-MS spectrum of the biodegradation product A of MC-LR.

**Figure 7:**
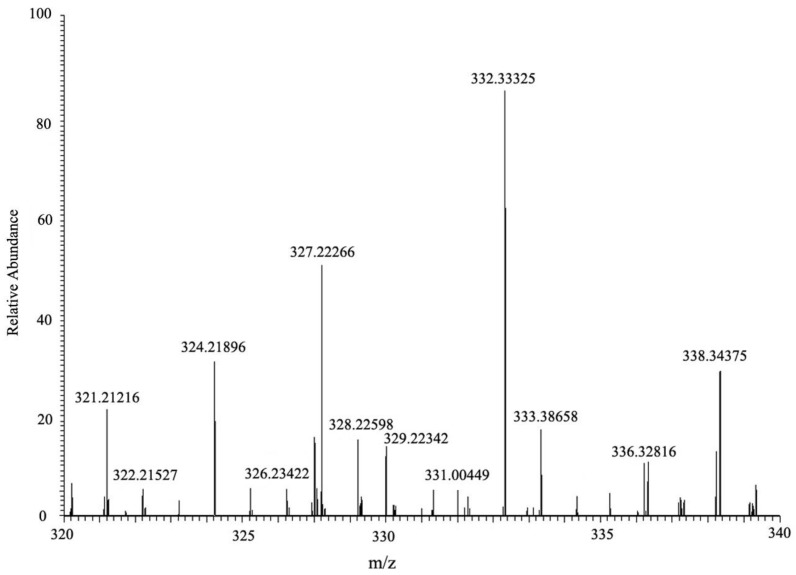
HPLC-ESI-MS spectrum of the biodegradation product B of MC-LR.

**Figure 8:**
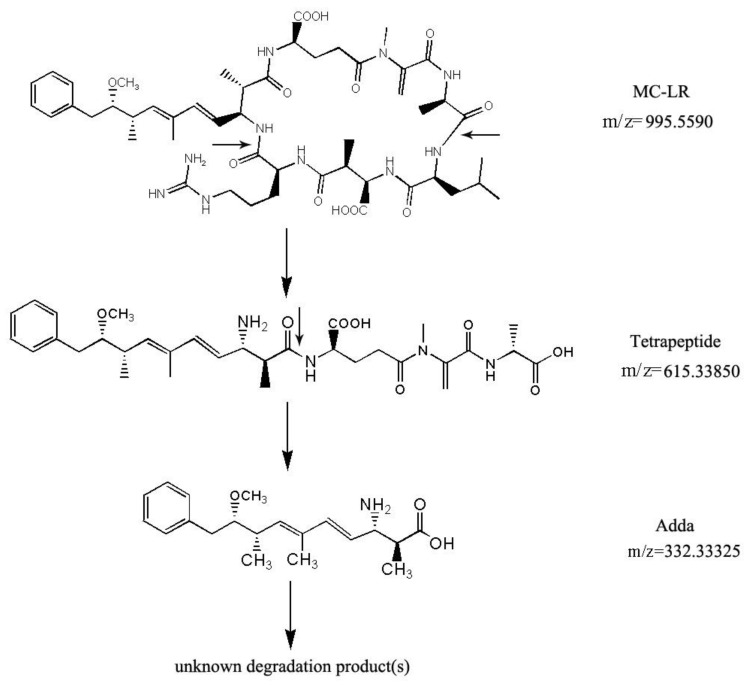
Putative degradation pathway of MC-LR and the formation of intermediate products (tetrapeptide and Adda) by YFMCD4. The small arrows indicate sites of peptide hydrolysis.

HPLC chromatograms of MC-LR and its degradation products are shown in Figure 5. Degradation of MC-LR by bacterial community YFMCD4 was tested in culture under the optimal conditions of 30 °C, pH 7, with 5 µg/mL of MC-LR concentration in the culture. HPLC chromatograms showed the retention time of MC-LR was 8.1 min (Figure 5a). The peak area of MC-LR decreased significantly after incubation and two main intermediate degradation products of MC (peak A and B) were apparent at 4 h (Figure 5b). The disappearance of all the peaks demonstrated complete catabolism of MC-LR and its degradation products by YFMCD4 in 10 h (Figure 5c). The degradation products peak A and B were further identified using the HPLC-ESI-MS, and exhibited accompanying ion at m/z 615.33850 (Figure 6) and m/z 332.33325 (Figure 7). The HPLC chromatograms and ions of peak A were identical to the tetrapeptide found by Bourne [24]. The ions of peak B were also identical to Adda which was the final MC degradation product of the *Sphingopyxis* C-1 [25] and the immediate degradation products of *Bordetella* sp. MC-LTH1 [3]. The degradation products indicate that the degradation pathway of YFMCD4 probably may be similar with that of *Bordetella* sp. MC-LTH1 [3] (Figure 8).

## 3. Discussion

Microbial biodegradation is an environment-friendly and effective treatment method to detoxify MC-LR in natural waters without potential harmful by-products. Some MC-degrading pure bacterial strains have been isolated and have their MC-LR-degrading rates reported [6,19,20,23]. For example, the single pure bacterial strain *Sphingomonas* sp. ACM-3962 (1.7 µg/(mL·day)) [26], LH21 (2.1 µg/(mL·day)) [27] and EMS (0.7 µg/(mL·day)) [28], *Ralstonia solanacearum* (9.4 µg/(mL·day)) [29], *Bordetella* sp. MC-LTH1 (7.4 µg/(mL·day)) [3], and *Stenotrophomonas* sp. MC-LTH2 (3 µg/(mL·day)) [18] have been studied. Until now, only a few MC-degrading bacteria mixed cultures have been obtained and investigated [19,30]. Cousins et al. [30] reported that a bacterial community showed the MC-degrading rate of 1.4 × 10^−3^ µg/(mL·day) while what kinds of bacteria existed in the community still needs to be studied. Ramani et al. [19] found a bacterial community containing two pure bacterial strains *Rhizobium* sp. DC7 and *Microbacterium* sp. DC8 degraded MC at 0.18 µg/(mL·day) while individual DC7 or DC8 could not degrade MC-LR, respectively. Tsao et al. [23] discovered a mixed culture with MC-degradation rate of 0.876 µg/(mL·day), which contained *Sphingomonas* sp., *Pseudoxanthomonas* sp., *Hyphomicrobium aestuarii*, *Sphingobium* sp., *Rhizobium* sp., *Steroidobacter* sp. and *Acinetobacter* sp. The bacterial community YFMCD4 containing *Alcaligenes* sp. and *Stenotrophomonas* sp. showed a higher degradation rate of MC-LR at 12 µg/(mL·day) compared with the single bacterial strain and most prior bacterial communities. These results confirmed that indigenous bacterial community always appeared to be more effective and suitable for degrading MC-LR than single pure bacterial strains, which is in accordance with the previous findings by Yang et al. [6], Ramani et al. [19] and Zhang et al. [29]. Our previous study [6] showed another natural bacterial community YFMCD1 including *Klebsiella* sp. or *Stenotrophomonas* sp. with the MC-degrading rate of 12 µg/(mL·day). In general, different bacterial communities owed different MC-degrading rates because they contained different pure bacterial species. However, it is interesting that the bacterial communities YFMCD4 and YFMCD1 exhibited the same highest MC-degrading rate, which means that different bacterial communities may have a similar MC-degrading rate although they consisted of different kinds of pure bacterial strains.

It was discovered that, the two single isolated bacteria strains when used independently degraded MC-LR at a much slower average rate of 0.96 × 10^−2^ µg/(mL·h) and 0.68 × 10^−2^ µg/(mL·h) than that of the bacterial community YFMCD4. This confirmed mixed culture may be more effective for degrading MC-LR than single bacterial strains. This may indicate the nutrient agar medium for isolating the bacterial strains YFMCD4-1 and YFMCD4-2 from bacterial community may not be good enough for isolating the highly effective microcystin-degrading bacteria. Therefore, it is better to analyze the structure of the bacterial community YFMCD4 using 16S rRNA gene high-throughput sequencing technique and then may isolate the pure highly effective dominant bacterial strains in the future if the high MC-degrading rates of the assemblages is driven by one particularly effective species.

The MC-degrading rates of bacterial community YFMCD4 were significantly affected by different temperatures, pH and MC-LR concentrations. The optimal conditions for degradation of MC-LR by YFMCD4 occurred at 30 °C, pH 7, and MC-LR concentration of 4 µg/mL or 5 µg/mL. Prior studies and our previous studies showed that these three factors play an important role in MCs degradation [18,31,32,33]. Park et al. [32] found the degradation rates were strongly dependent on temperature and the MC-degrading rate was very slow at 5 °C while the maximum degradation rate occurred at 30 °C. Ramani et al. [19] found temperature has some effects on MC-LR degradation and the optimal degradation rate was achieved at 26 °C. Yang et al. [6] also discovered that the degradation rates changed when the temperature varied, and the best temperature for MC-LR degradation was 30 °C. It was necessary to investigate the influence of pH on MC-LR degradation activities of bacteria because the pH of water bodies varies during cyanobacterial blooms [34]. The highest ability of YFMCD4 to degrade MC-LR under neutral environment suggested that YFMCD4 may contain MC-degrading enzymes which are different from alkali-tolerant protease secreted by *Sphigopyxis* sp. C-1 [34].

Further work needs to determine the practical application of the bacterial community YFMCD4 in water bodies contaminated with MCs. Biological sand filters embedded with MC-degrading bacterium *Sphingomonas* sp. MJ-PV removed MC-LR successfully, which offered an effective and cost-efficient treatment process [35,36]. Thus, an in situ effective degradation of MC-LR might be achieved by implementing biological filtration embedded with YFMCD4 [3,35,36].

Two kinds of intermediates of MC-LR degradation were identified as the linearized MC-LR and a tetrapeptide in the previous studies [3,18,24,37]. Moreover, the intact Adda was isolated and identified from the final MC-LR degradation products using *Sphingomonas* sp. B-9 [37]. In this study, two intermediates, Adda and a tetrapeptide, appeared when YFMCD4 degraded MC-LR, and the Adda finally disappeared. The results therefore showed, the MC-degrading mechanism of the bacterial community YFMCD4 is different from that of the previous bacteria *Sphingomonas* sp. B-9 [37] and ACM-3962 [26] as well as *Sphigopyxis* sp. C-1 [34]. The MC-degrading mechanism of the bacterial community YFMCD4 is also possibly different from that of the bacterial community YFMCD1 due to the absence of tetrapeptide in the MC-degrading products using YFMCD1. It is well known that Adda is essential for the biological activities of MC-LR [35]. In this study, the Adda was completely degraded, which suggested that the bacterial community YFMCD4 has the capacity of detoxifying MC-LR [3,6,18]. The degradation products of Adda needed to be further isolated and clarified, and it is important to investigate the practical MC-degrading effects of YFMCD4 when it is applied into different kinds of water polluted by MC-LR in the future.

## 4. Conclusions

A novel native effective MC-degrading bacterial community YFMCD4 was obtained from Lake Taihu, which is one of the highest effective MC-degrading bacterial communities until now. Two pure bacterial strains *Alcaligenes faecalis* YFMCD4-1 and *Stenotrophomonas acidaminiohila* YFMCD4-2 were isolated from the bacterial community YFMCD4. These isolated bacteria strains independently degrade MC-LR at a slower average rates of 0.96 × 10^−2^ µg/(mL·h) and 0.68 × 10^−2^ µg/(mL·h), respectively. The degradation rate of MC-LR by bacterial community YFMCD4 significantly influenced by various pH, temperature and MC-LR concentrations, and the highest rate reached 0.5 µg/(mL·h) under the condition of 30 °C and pH 7 with MC-LR concentrations of 4 or 5 µg/mL. Two intermediates of tetrapeptide and Adda existed in the MC-degrading products, and the Adda can also be completely degraded by the bacterial community YFMCD4. Therefore, the bacterial community YFMCD4 can completely degrade MC-LR effectively under some conditions and has a great potential for the bioremediation of water polluted by MC-LR.

## 5. Materials and Methods

### 5.1. Materials and Reagents

MC-LR was purchased from Alexis Corporation (Lausen, Switzerland) and stored at −20 °C (purity ≥95%). Formic acid and methanol used for high-performance liquid chromatography (HPLC) and ultra-high resolution LTQ OrbitrapVelos Pro ETD mass spectrometry equipped with electrospray ionization interface (HPLC-ESI-MS) analysis were purchased from Dikma Technology Incorporation (Foothill Ranch, CA, USA). The mineral salt medium (MSM) for bacterial culture, acquisition and MC-LR removal was prepared as previous study [3,6,18].

### 5.2. Acquisition of a Novel Native Bacterial Community YFMCD4 and Bacterial Strains from the Bacterial Mixed Culture

Five gram of wet sludge sample was collected from Lake Taihu and suspended in 45 mL MSM. A novel MC-degrading bacterial community was obtained and designated as YFMCD4 in 24 days under the conditions previously described by Yang et al. [6]. The bacterial community YFMCD4 serially diluted with sterile MSM and 0.1 mL of each dilution were inoculated onto nutrient agar (2% agar) plates. Two pure bacterial strains named YFMCD4-1 and YFMCD4-2 were isolated.

16S rRNA gene fragments of YFMCD4-1 and YFMCD4-2 were amplified using PCR with the universal primers 5’-AGAGTTTGATCMTGGCTCAG-3’ and 5’-TACGGYTACCTTGTTACGAACTT-3’) under the conditions previously described by Yang et al. [4]. The PCR products were sequenced by the Sangon Biotech Incorporation located in Shanghai, China. Nucleotide sequences comparisons were conducted using the National Center for Biotechnology Information (NCBI) database (http://www.ncbi.nlm.nih.gov/BLAST). The program ClustalW 2.1 was applied to align the entire similar 16S rRNA gene sequences downloaded from the NCBI database. Phylogenetic trees were successfully generated via the neighbor-joining method using the MEGA software designed by Tamura et al. [38].

### 5.3. MC-LR Degradation by Bacterial Community YFMCD4 and the Isolated Bacteria

To study MC-LR-degrading ability of YFMCD4, the bacterial community YFMCD4 was cultured with MC-LR under different incubation conditions including different temperatures at 20 °C, 30 °C or 40 °C, at MC-LR concentrations 1, 2, 3, 4, or 5 µg/mL, and at pH 3, 5, 7, 9, or 11. Fifteen microliter samples were withdrawn at intervals and centrifuged (12,000× *g*, 15 min, 4 °C) for monitoring the concentrations of MC-LR in all the samples using HPLC. All the experiments were in triplicate with bacterial free samples serving as the control.

To investigate the pure bacteria strains, YFMCD4-1 and YFMCD4-2 were cultured in liquid nutrient broth (NB) medium for two days and transferred to MSM medium containing 2 mg/L MC-LR at 30 °C, 120 rpm, respectively. The bacterial cells were harvested by centrifugation (5000× *g*, 15 min, 4 °C) and suspended into MSM containing standard MC-LR. In the experiment, 15 μL of the sample was taken, centrifuged (12,000× *g*, 15 min, 4 °C) and determined the MC-LR concentration by HPLC.

### 5.4. Analysis of MC-LR and its Degrading Products

The Agilent 1100 HPLC machine with a Zorbax Extend C_18_ column (4.6 × 150 mm, 5 µm, Agilent, Palo Alto, CA, USA) and a variable wavelength detector (VWD) set at 238 nm was employed for analyzing MC-LR and degradation products. The mobile phase was a mixture of 0.1% trifluoroacetic acid aqueous solution and methanol (37:63, *v/v*) set at a flow rate of 0.8 mL/min, injection volume 10 µL and column temperature 40 °C.

The MC-degrading products were identified by HPLC-ESI-MS. Both the auxiliary and sheath gases were nitrogen at a flow rate of 30 and 5 psi, respectively. The dry gas temperature was set at 350 °C and nebulizer pressure at 45 psi. Spectra were recorded in positive modes at a spray voltage of 3.5 kV.

## Supplementary Materials

The following are available online at http://www.mdpi.com/2072-6651/10/9/363/s1, Figure S1: The degradation activities of MC-LR by YFMCD4-1 and YFMCD4-2.
