# Supplementary material for: Removal of Microcystin-LR by a Novel Native Effective Bacterial Community Designated as YFMCD4 Isolated from Lake Taihu
Source: Toxins (Basel). 2018 Sep 8;10(9):363. doi: 10.3390/toxins10090363 (PMC6162702; doi:10.3390/toxins10090363)
Supplement: Supplementary file 1 [file toxins-10-00363-s001.pdf]

SupplementaryMaterials: Removal of Microcystin-LR by a Novel Native Effective Bacterial Community Designated as YFMCD4 Isolated from Lake Taihu

Fei Yang [1,2,3,*], Jian Guo [1], Feiyu Huang [1], Isaac Yaw Massey [1], Ruixue Huang [1,*], Yunhui Li [2], Cong Wen [1], Ping Ding [1], Weiming Zeng [4] and Geyu Liang [2]

[1] Department of Occupational and Environmental Health, Xiangya School of Public Health, Central South University, 110 Xiangya Road, Changsha 410078, China; guojianph@csu.edu.cn (J.G.); huangfeiyu@csu.edu.cn (F.H.); robert@csu.edu.cn (I.Y.M.); wencong941017@csu.edu.cn (C.W.); yixp176911007@csu.edu.cn (P.D.)

[2] Key Laboratory of Environmental Medicine Engineering, Ministry of Education, School of Public Health Southeast University, Nanjing 210009, China; yhli@seu.edu.cn (Y.L.); gyliang@seu.edu.cn (G.L.)

[3] Key laboratory of Hunan Province for Water Environment and Agriculture Product Safety, Central South University, Changsha 410083, China;

[4] Key Laboratory of Biometallurgy, Ministry of Education, School of Minerals Processing and Bioengineering, Central South University, Changsha 410083, China; hyacinth_hai@csu.edu.cn (W.Z.)

* Correspondence: phfyang@csu.edu.cn (F.Y.); zhengshl@csu.edu.cn (R.H.); Tel./Fax: +86-731-84805460 (F.Y. & R.H.)

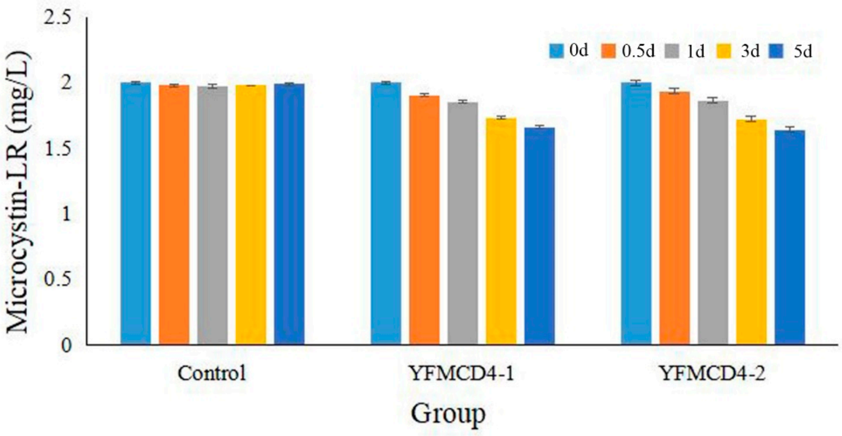

Figure S1. The degradation activities of MC-LR by YFMCD4-1 and YFMCD4-2.
